# Structural Neuroimaging Markers of Cognitive Decline in Parkinson's Disease

**DOI:** 10.1155/2016/3217960

**Published:** 2016-04-14

**Authors:** Alexandru Hanganu, Oury Monchi

**Affiliations:** ^1^Department of Clinical Neurosciences and Department of Radiology, University of Calgary, Calgary, AB, Canada T2N 1N4; ^2^Hotchkiss Brain Institute, University of Calgary, Calgary, AB, Canada T2N 4N1; ^3^Centre de Recherche, Institut Universitaire de Gériatrie de Montréal, Université de Montréal, Montréal, QC, Canada H3W 1W5; ^4^McGill University, Montreal, QC, Canada H3A 0G4; ^5^Department of Radiology, Faculty of Medicine, University of Montreal, Montreal, QC, Canada H3T 1J4

## Abstract

Cognitive impairment in patients with Parkinson's disease is a major challenge since it has been established that 25 to 40% of patients will develop cognitive impairment early in the disease. Furthermore, it has been reported that up to 80% of Parkinsonian patients will eventually develop dementia. Thus, it is important to improve the diagnosing procedures in order to detect cognitive impairment at early stages of development and to delay as much as possible the developing of dementia. One major challenge is that patients with mild cognitive impairment exhibit measurable cognitive deficits according to recently established criteria, yet those deficits are not severe enough to interfere with daily living, hence being avoided by patients, and might be overseen by clinicians. Recent advances in neuroimaging brain analysis allowed the establishment of several anatomical markers that have the potential to be considered for early detection of cognitive impairment in Parkinsonian patients. This review aims to outline the neuroimaging possibilities in diagnosing cognitive impairment in patients with Parkinson's disease and to take into consideration the near-future possibilities of their implementation into clinical practice.

## 1. Introduction

Parkinson's disease (PD) is a movement disorder and the second most frequent chronic neurodegenerative disease, affecting up to 2 percent among persons older than 65 years [1] and nearly 10% of people older than 80 years [2]. Cognitive deficits were consistently reported in patients with PD for measures of executive function and working memory, suggesting dysfunctional frontostriatal brain circuitry [3–5]. It has been established that up to 40% of patients with PD present with mild cognitive impairment (MCI) early in the disease [6]. MCI is defined as a cognitive deficit commonly quantified as a performance level 1-2 SDs below the population mean in one or more cognitive domains [7]. Some studies suggested that PD patients with MCI (PD-MCI) have an increased risk of developing dementia compared with patients with normal cognition (PD-NC) [8–10]. A prospective study of 8-year follow-up reported that 78,2% of PD patients eventually develop dementia [11], and from the PD-MCI group up to 62% of patients converted to dementia over a 4-year period, compared with 20% of PD-NC patients [10, 12, 13]. Thus, avoiding dementia becomes a key part in preserving an active life for PD patients and early MCI diagnosis allows us to take the necessary steps for achieving that goal.

The diagnosis of MCI and dementia in PD remains still clinical and neuroimaging techniques can only be used as supportive measures. Several neuroimaging parameters have been described and can be used to analyze the changes in gray matter structure. Cortical thickness and cortical surface area have been proposed as useful measures to analyze the cortical gray matter morphology, as they have the advantage of providing a direct quantitative index [14, 15]. A third parameter, the gray matter volume, can be measured in both cortical and subcortical structures. Cortical thickness is measured as the closest distance from the gray/white matter boundary to the gray/cerebrospinal fluid boundary. To measure the cortical surface area, the cortex is arranged in a triangular grid and the final value of the surface area is calculated by measuring the area of each triangle of the midsurface and assigning one-third of this area to each of its three vertices (i.e., the area assigned to each vertex is one-third of the total area of all triangular facets adjoining it). The cortical gray matter volume is a product of thickness and surface area [16] while the volumes of subcortical gray matter structures represent the volume of the three-dimensional space, based on the automatic or semimanually defined regions of interest.

Based upon the previous description that neurons within the cerebral cortex are organized into ontogenetic columns that run perpendicular to the brain surface [17], it has been suggested that measurement of cortical thickness is linked with the number of cells within a column while surface area relates directly to minicolumn number and spacing [15, 18, 19]. Thus, the concept of cortical thickness reflects the arrangement of neurons and neuropil in a biologically and topologically meaningful way; cortical surface area assesses the local cortical folding while the changes in gray matter volume may reflect changes in either thickness, surface area, or both [15, 16, 19]. Other studies suggested that gray matter volume correlates more with surface area, because the regional area is measured on the surface between adjacent landmarks, giving a higher quadratic weight to tangential (horizontal) than to vertical (radial) distances [20]. Due to this criterion, surface area tends to have more variability and thus gray matter volume also has greater variability [20], implying that gray matter volume-based measurement techniques might have a lower reliability compared with cortical thickness-based measurement techniques.

From a morphological point of view, cortical thickness does not directly relate to neuronal loss or neuronal density. It has been shown that over time some brain regions (lateral and mesial prefrontal and inferior parietal) maintain a relatively constant cortical thickness and neuronal density, whereas in other regions (all remaining brain regions) neuronal density linearly decreased with increased thickness [21]. Thus, lower cortical thickness does not necessarily mean neuronal loss, but rather loss of neuronal and dendritic architecture, for example, reduced size of neuronal cell body, reduced dendritic arborization, or the loss of presynaptic terminals [22]. Additionally, local surface area might reflect the state of the underlying white matter fibres, as more tension or shrinkage of these fibres could lead to deeper sulci and extended cortical surface area. Thus, cortical surface area can also indirectly reflect white matter tract damage [23].

Some current neuroimaging software programs have the potential to be used for automatic integration in a pipeline and assessment of gray matter measurements on an individual level. These include Freesurfer (http://surfer.nmr.mgh.harvard.edu/) [24], voxel-based morphometry (VBM) analyses using the FMRIB Software Library (FSL) (http://www.fmrib.ox.ac.uk/fsl/fslvbm) [25], Statistical Parametric Mapping (SPM) analyses in MATLAB (MathWorks, Inc., Natick, MA, USA) using the VBM toolbox (http://www.fil.ion.ucl.ac.uk/spm/) [26], and the SurfStat toolbox (http://www.math.mcgill.ca/keith/surfstat/) [27] in MATLAB. This brings the possibility for defining brain structural markers for certain pathologies and for measuring the cortical parameters on an individual level, thus opening the opportunity for a quick estimation of the risk for developing the pathology and for assessing its progression over time. One additional approach to do this on individual level is to use automatic learning algorithms such as Support Vector Machines, which allow both the definitions of new patients' groups as well as single-patient classifications into those groups [28]. Considering that in a clinical setting the costs for performing a structural T1 magnetic resonance imaging (MRI) sequence are lower compared to a diffusion weighted image (DWI) and that analyzing the gray matter parameters of cortical thickness and cortical surface area deformation can be easily performed without manual interventions compared with analyses of white matter parameters which need manual adjustments at more stages, currently it seems more feasible to implement an automated pipeline based on structural gray matter changes. Thus, the present review will focus mainly on the brain structural markers of cognitive decline in patients with Parkinson's disease with emphasis on the gray matter markers.

Studies in the last decade analyzed the patterns of brain anatomical changes in Parkinsonian patients with and without dementia and in comparison to healthy controls. First, it is important to note that in several cross-sectional and longitudinal studies in which PD patients were distributed in groups based on their cognitive status, PD-NC patients did not demonstrate significant brain atrophy compared with healthy controls [29–31]. Second, PD patients with dementia (PDD) had significant anatomical changes compared with nondemented PD patients (NDPD, which includes both PD-NC and PD-MCI) and healthy controls. Burton et al. [32] reported that PDD have reduced gray matter volume compared to healthy controls in the hippocampus, parahippocampal gyrus, occipital lobe, right frontal lobe, and left parietal lobes. In addition, when compared with NDPD patients, PDD had more gray matter atrophy in the occipital lobe, suggesting that in PDD the atrophy pattern extends to temporal, occipital, and subcortical areas, but occipital atrophy being the only difference between the groups. By contrast, other groups that analyzed the gray matter volume changes between PDD and PD using VBM reported significant differences only in the left superior temporal gyrus and right hippocampus [33] or in the dorsolateral prefrontal cortex (DLPFC), anterior cingulate gyrus, temporal lobe, and subcortical regions, hippocampus, thalamus, and caudate nucleus [34]. In continuation to this pattern, another VBM study examined the gray matter changes in PDD compared with PD-NC and PD-MCI patients. Results showed gray matter reductions in PD-MCI patients in the left middle frontal gyrus, precentral gyrus, left superior temporal lobe, and right inferior temporal lobe compared to PD-NC patients, while the PDD group was reported to have reduced gray matter volume in the bilateral frontal, temporal, parietal lobes and the limbic region (medial occipital) compared to PD-NC and PD-MCI combined [35]. Our group analyzed the cortical changes over time and reported a higher rate of cortical thinning in PD-MCI patients in the supplementary motor area (SMA), superior temporal gyrus, superior parietal region, and medial occipital cortex [29]. A recent longitudinal study on a bigger group of Parkinsonian patients reported that PD-MCI and PD-NC did not have significant differences in regional cortical thickness at baseline, while after 18 months the PD-MCI group demonstrated widespread cortical thinning in the bilateral pre-SMA, right SMA, and left superior temporal gyrus in comparison with PD-NC patients [30]. Interestingly both longitudinal studies reported the atrophy of nucleus accumbens in PD-MCI patients in comparison with PD-NC as well as healthy controls [29, 30]. Furthermore, PD-MCI patients showed a correlation between the Montreal Cognitive Assessment scale scores and cortical thinning in the left postcentral gyrus, middle and inferior temporal gyri, and right fusiform gyrus in one study [29], as well as left superior frontal, orbitofrontal, inferior parietal cortices and left fusiform and right parahippocampal gyri in another study [30]. Hence, several cortical and subcortical regions might have the potential to be considered as markers of cognitive impairment associated neurodegeneration.

## 2. Frontal Lobe

**Figure 1:**
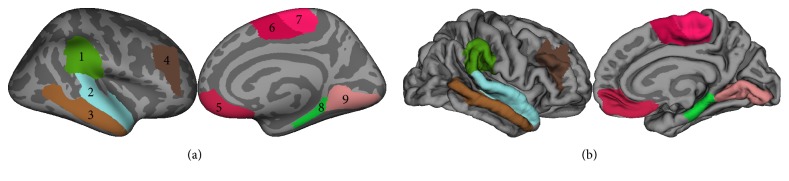
Cortical regions discussed in the present review. Cluster nr 1: supramarginal gyrus; 2: superior temporal gyrus; 3: middle temporal gyrus; 4: dorsolateral prefrontal cortex (DLPFC); 5: ventromedial prefrontal cortex (VMPFC); 6: presupplementary motor area (pre-SMA); 7: supplementary motor area (SMA); 8: parahippocampal gyrus; 9: lingual area. Regions were drawn on the fsaverage subject in FreeSurfer 5.3 based on the Desikan atlas [82]. An inflated right hemisphere is presented (a) and the version outlining the gyri and sulci (b). SMA and pre-SMA were drawn based on the Brodmann atlas in FreeSurfer. The DLPFC region was drawn over Brodmann areas 9 and 46 [83] with the dorsal and ventral borders at the bottom of the superior and inferior frontal sulci, rostral border at the anterior termination of the olfactory sulcus [84], and the caudal border at the limit of Brodmann areas 8 and 9 [83]. Image created with GIMP Image Manipulation Program 2.8.16.

SMA and pre-SMA are the primary potential candidates as structural neuroimaging markers specific for MCI in PD patients (Figure 1, clusters 6 and 7). Bilateral SMA and pre-SMA were shown to have faster rates of cortical thinning over time in PD-MCI patients compared with PD-NC and healthy controls in longitudinal studies [29, 30]. Cross-sectional studies revealed structural gray and white matter changes in PD when compared with healthy controls. PD-MCI patients were shown to have cortical thinning [36] and decreased fractional anisotropy (a diffusion imaging measure thought to reflect fiber density, axonal diameter, and myelination) in the anterior superior longitudinal fasciculus (which connects the SMA and DLPFC with superior and medial parietal cortex) [37] while NDPD patients were reported to have SMA cortical gray matter thinning [38], decreased white matter fractional anisotropy [39], and connectivity changes (the SMA-cerebellum connection was inhibitory in PD and excitatory in healthy controls [40]). Poorer backward digit span test of the Wechsler Adult Intelligence Scale (a measure of short-term memory) was shown to correlate with lower fractional anisotropy in the paracentral lobule (the anterior portion of which refers to SMA) [41]. Pre-SMA showed hypometabolism associated with cognitive deficits [42]. Moreover, in our previous study in NDPD, cortical thinning and area enlargement in SMA showed significant positive correlation with duration of disease [43] while another pathological study has disclosed significant loss of corticocortical projecting pyramidal neurons in the pre-SMA without intraneuronal inclusions [44]. The results of the later studies might have been driven by the PD-MCI group.

Other frontal clusters were also reported. Specifically, cross-sectional analysis showed reduced gray matter volume in PDD compared with NDPD in the frontal lobes bilaterally [35] and PDD versus healthy controls had gray matter atrophic changes in the DLPFC [34] and anterior cingulate [33, 34]. Other studies reported gray matter reductions in PD-MCI in the middle frontal gyrus and precentral gyrus compared with PD-NC patients [35] and diminished cortical thickness in the ventromedial prefrontal cortex (VMPFC) and premotor cortices when compared with healthy controls [36, 43]. NDPD patients had reduced gray matter volume compared with healthy controls in the superior, middle, and inferior frontal gyri [32]. Longitudinal analyses in PD-MCI compared with healthy controls reported cortical atrophy in the VMPFC, DLPFC, and premotor cortices [29, 30]. On the other hand, several studies reported no significant differences in the DLPFC, VMPFC, and anterior cingulate between NDPD and healthy controls [33, 34] or NDPD and PDD [33]. The distinct results can be due to different techniques for measuring the gray matter changes, different smoothing thresholds, different methods of creating the average subject for the groups, and different methods of accounting for confounding factors such as age, disease duration, education, and levodopa dosages [45].

The presence of some of these frontal clusters is in line with previously described impairment in the frontostriatal brain circuitry in PD patients [3–5, 46–48] according to which basal ganglia control the functioning of the frontal regions [49]. Two of these circuits include the lateral orbitofrontal cortex (part of the VMPFC) and the “motor circuit” that is primarily directed to the precentral motor fields [50, 51]. Lesioned prefrontal cortex [52] and lesions of the VMPFC induce the inability to weigh cost versus benefits in the Iowa gambling task [53, 54] and can cause profound changes in emotional and social behaviours, including impairments in certain aspects of decision making [55, 56]. Such functional impairments have been described in PD patients; they are impaired at making choices that require learning from trial and error [57, 58] and in decision making during explicit gambling situations [59]. However, some frontal clusters are reported by some studies but not by others and tend to appear in the contrasts that compare PD patients with healthy controls but do not appear in the contrasts between different cognitive profiles of PD (PDD versus NDPD, PDD versus PD-MCI, and PD-MCI versus PD-NC). Hence, we can distinguish three groups of clusters in the frontal lobe: (1) clusters that have structural changes due to the presence of PD and have little associations with cognitive impairment (the motor and premotor cortices), (2) clusters that show some association with cognitive impairment but can be considered reliable only with the presence of additional neuropsychological testing (DLPFC, VMPFC) (Figure 1, clusters 4 and 5), (3) and clusters that seem to be associated directly with cognitive impairment and can be reliable structural markers that indicate the presence of cortical changes due to cognitive impairment in PD (SMA and pre-SMA).

## 3. Temporal Lobe

A large number of studies reported temporal lobe changes associated with MCI in PD patients. Cross-sectional studies reported cortical atrophy in PD-MCI [36] as well as PDD patients [32]. Specifically, PD-MCI were reported to have changes in the medial temporal lobe with thinner parahippocampal and fusiform cortices [31, 60] as well as increased surface area [43] when compared to PD-NC and greater cortical thinning in the superior and middle temporal gyri in early PD patients compared with healthy controls [36, 61]. Temporal lobe changes were shown to have a positive correlation with cognitive scores [29, 30] and a negative correlation with the duration of disease in PD-MCI but not in PD-NC [43]. Longitudinal studies in PD-MCI patients reported a higher rate of cortical atrophy and an increased percentage of cortical thinning over time in the superior temporal gyrus and temporal pole in PD-MCI compared to PD-NC or healthy controls [29, 30]. Additionally, deficits in neuropsychological memory tasks likely relying on the medium temporal lobe were reported to be more associated with incident dementia compared with the frontally based planning and working memory deficits [13]. Studies in non-PD populations reported that reduced medial and lateral temporal lobe atrophy was also present in MCI patients who converted to Alzheimer's disease [14, 62]. Furthermore, temporal lobe atrophy was shown to be a powerful and independent predictor of conversion to dementia in relatively young MCI patients [63]. Overall, this would indicate that temporal lobe atrophy (specifically the parahippocampal gyrus, superior and middle temporal gyri) is strongly linked with cognitive impairment in both PD and non-PD populations (Figure 1, clusters 2, 3, and 8). This also highlights the importance of distinguishing between cognitive domains (e.g., amnestic versus nonamnestic) in PD-MCI in the early detection of patients who are likely to develop dementia rapidly.

## 4. Medial Occipital Cortex

A similar interpretation can be attributed to changes in the medial occipital lobe. Specifically the lingual region (Figure 1, cluster 9) was reported to have significant structural changes in PD-MCI patients compared with PD-NC. Gray matter loss in the lingual cortex has been reported by cross-sectional [36] and longitudinal studies [29]. This region also showed lower surface area, negative correlations with duration of disease [43], and cognitive scores over time [30]. In PDD patients bilateral occipital lobe atrophy was reported using VBM [32]. Metabolic changes were also described in the medial occipital lobe: increased hypoperfusion (compared with other brain regions) [64, 65] and a greater cerebral glucose metabolic rate reduction (compared with healthy controls) [66]. Yet, another longitudinal study did not find significant cortical changes over time in PD-MCI patients in the occipital lobe [30]. From a functional point of view, the occipital structural changes in PD can be associated with the presence of hallucinations, since up to 40% of patients with PD have hallucinations and they are almost exclusively visual [67–70]. Previous studies reported a correlation between gray matter volume reduction and visual hallucinations in PD [71] as well as a dysfunction of the ventral visual pathway in PD patients with visual hallucinations and cognitive impairment [72]. Considering the above facts, it is still not clear whether occipital structural changes are linked with cognitive impairment or they reflect a more global cortical degeneration. Probably occipital lobe atrophy in patients who were diagnosed with PD can be considered as a marker of cognitive decline only if visual hallucinations are present.

## 5. Subcortical Structures

Anatomical neuroimaging cross-sectional studies in NDPD reported volume changes in subcortical gray matter structures in comparison to healthy controls. Cross-sectional and longitudinal studies reported lower volumes in the caudate nucleus, putamen, thalamus [32, 34, 73], hippocampus [30, 31, 74, 75], and nucleus accumbens [29, 30, 75, 76]. Further longitudinal analysis outlined that over time volume shrinkage in these structures is present only in PD-MCI patients and are absent in PD-NC [29], suggesting that volume parameters of subcortical structures in PD-NC are closer to the parameters of healthy controls, while volume losses are more specific for appearance of cognitive impairment. In support for this result, volume loss was reported in the nucleus accumbens only in PD-MCI patients [29, 30], which was in line with the dopamine depletion theory in PD, according to which dopamine loss in PD progresses from the dorsal striatum (specifically caudate nucleus) to the ventral striatum (nucleus accumbens) and in early PD the dorsal striatum is severely depleted while the ventral striatum is relatively intact [77, 78]. Hence, nucleus accumbens volume loss in PD-MCI patients can be considered as a marker for a higher level of dopamine depletion compared to PD-NC patients and a marker for the establishment of cognitive impairment.

## 6. Parietal Lobe

The structural changes in the parietal lobe have not been conclusive. The medial parietal cortex was reported to have decreased cortical thickness in one cross-sectional study [60]. The postcentral gyrus was reported with increased surface area cross-sectionally [43] and cortical gray matter shrinkage over time in PD-MCI patients compared with PD-NC or healthy controls [30]. A third cluster, right supramarginal (Figure 1, cluster 1), was shown to have a significant larger surface area in NDPD patients compared with healthy controls [38] and cortical shrinkage over time in PD-MCI compared to healthy controls [30]. Additionally, the right supramarginal and angular regions showed a positive correlation between cortical thickness and duration of disease in PD-MCI patients compared to PD-NC, thus showing a clear relation with cognitive decline in PD [43]. But most of these structural changes were not replicated by other studies. On the other hand, incident dementia was reported to be more specific for PD patients with deficits in neuropsychological tasks with a temporal and parietal lobe basis [13]. Thus, a marker in the parietal lobe could be considered reliable only if it was reported by several studies and in a big cohort. The supramarginal gyrus is one such cluster. Indeed, this region is involved in visual word recognition [79], intonation of speech [80], and the word's sounds [81] and in PD patients both speech production and self-monitoring of voiced speech are altered [80]. Nevertheless, future studies are needed in order to confirm the reliability of the supramarginal gyrus.

## 7. Conclusion

The diagnosis of MCI in PD should remain to be mainly clinical but neuroimaging techniques should be used as supportive findings. We suggest that several reliable MRI markers have already been distinguished and they can be used in order to predict cognitive decline in PD patients. Markers with an increased reliability that should be considered include the SMA and nucleus accumbens, along with atrophy in the temporal lobe (specifically parahippocampal, superior temporal, and middle temporal gyri) and medial occipital lobe (specifically the lingual area). Structural changes in other regions such as the DLPFC and VMPFC may also be markers of cognitive decline, but further studies are required to find out to what degree.

We suggest that current techniques could allow for the development of automated pipelines that measure the parameters of gray matter changes and that might be implementable on clinical MRI systems. Such approaches have the potential to be used in the future at the individual level after the creation of highly reliable average brains of PD patients with normal cognition and PD patients with MCI.

## References

[1] De Rijk M. C., Tzourio C., Breteler M. M. B. (1997). Prevalence of parkinsonism and Parkinson's disease in Europe: the EUROPARKINSON collaborative study. European Community Concerted Action on the Epidemiology of Parkinson's disease. *Journal of Neurology Neurosurgery & Psychiatry*.

[2] Von Campenhausen S., Bornschein B., Wick R. (2005). Prevalence and incidence of Parkinson's disease in Europe. *European Neuropsychopharmacology*.

[3] Lewis S. J. G., Dove A., Robbins T. W., Barker R. A., Owen A. M. (2003). Cognitive impairments in early Parkinson's disease are accompanied by reductions in activity in frontostriatal neural circuitry. *The Journal of Neuroscience*.

[4] Monchi O., Petrides M., Mejia-Constain B., Strafella A. P. (2007). Cortical activity in Parkinson's disease during executive processing depends on striatal involvement. *Brain*.

[5] Owen A. M. (2004). Cognitive dysfunction in Parkinson's disease: the role of frontostriatal circuitry. *Neuroscientist*.

[6] Aarsland D., Kurz M. W. (2010). The epidemiology of dementia associated with Parkinson disease. *Journal of the Neurological Sciences*.

[7] Litvan I., Goldman J. G., Tröster A. I. (2012). Diagnostic criteria for mild cognitive impairment in Parkinson's disease: movement disorder society task force guidelines. *Movement Disorders*.

[8] Emre M., Aarsland D., Brown R. (2007). Clinical diagnostic criteria for dementia associated with Parkinson's disease. *Movement Disorders*.

[9] Kehagia A. A., Barker R. A., Robbins T. W. (2010). Neuropsychological and clinical heterogeneity of cognitive impairment and dementia in patients with Parkinson's disease. *The Lancet Neurology*.

[10] Williams-Gray C. H., Foltynie T., Brayne C. E. G., Robbins T. W., Barker R. A. (2007). Evolution of cognitive dysfunction in an incident Parkinson's disease cohort. *Brain*.

[11] Aarsland D., Andersen K., Larsen J. P., Lolk A., Kragh-Sørensen P. (2003). Prevalence and characteristics of dementia in Parkinson disease: an 8-year prospective study. *Archives of Neurology*.

[12] Janvin C. C., Larsen J. P., Aarsland D., Hugdahl K. (2006). Subtypes of mild cognitive impairment in Parkinson's disease: progression to dementia. *Movement Disorders*.

[13] Williams-Gray C. H., Evans J. R., Goris A. (2009). The distinct cognitive syndromes of Parkinson's disease: 5 year follow-up of the CamPaIGN cohort. *Brain*.

[14] Lerch J. P., Evans A. C. (2005). Cortical thickness analysis examined through power analysis and a population simulation. *NeuroImage*.

[15] Parent A., Carpenter M. B. (1995). *Human Neuroanatomy*.

[16] Rimol L. M., Nesvåg R., Hagler D. J. (2012). Cortical volume, surface area, and thickness in schizophrenia and bipolar disorder. *Biological Psychiatry*.

[17] Mountcastle V. B. (1997). The columnar organization of the neocortex. *Brain*.

[18] Chance S. A., Casanova M. F., Switala A. E., Crow T. J. (2008). Auditory cortex asymmetry, altered minicolumn spacing and absence of ageing effects in schizophrenia. *Brain*.

[19] Rakic P. (1988). Specification of cerebral cortical areas. *Science*.

[20] Winkler A. M., Kochunov P., Blangero J. (2010). Cortical thickness or grey matter volume? The importance of selecting the phenotype for imaging genetics studies. *NeuroImage*.

[21] La Fougère C., Grant S., Kostikov A. (2011). Where in-vivo imaging meets cytoarchitectonics: the relationship between cortical thickness and neuronal density measured with high-resolution [18F]flumazenil-PET. *NeuroImage*.

[22] Freeman S. H., Kandel R., Cruz L. (2008). Preservation of neuronal number despite age-related cortical brain atrophy in elderly subjects without Alzheimer disease. *Journal of Neuropathology and Experimental Neurology*.

[23] Van Essen D. C. (1997). A tension-based theory of morphogenesis and compact wiring in the central nervous system. *Nature*.

[24] Fischl B., Dale A. M. (2000). Measuring the thickness of the human cerebral cortex from magnetic resonance images. *Proceedings of the National Academy of Sciences of the United States of America*.

[25] Douaud G., Smith S., Jenkinson M. (2007). Anatomically related grey and white matter abnormalities in adolescent-onset schizophrenia. *Brain*.

[26] Ashburner J., Friston K. J. (2000). Voxel-based morphometry-The methods. *NeuroImage*.

[27] Worsley K. J., Marrett S., Neelin P., Vandal A. C., Friston K. J., Evans A. C. (1996). A unified statistical approach for determining significant signals in images of cerebral activation. *Human Brain Mapping*.

[28] Forkert N. D., Sedlacik J., Boelmans K. Fast and fully automatic differentiation of patients with idiopathic Parkinsonian syndrome and progressive supranuclear palsy using T1-weighted MRI datasets.

[29] Hanganu A., Bedetti C., Degroot C. (2014). Mild cognitive impairment is linked with faster rate of cortical thinning in patients with Parkinson's disease longitudinally. *Brain*.

[30] Mak E., Su L., Williams G. B. (2015). Baseline and longitudinal grey matter changes in newly diagnosed Parkinson’s disease: ICICLE-PD study. *Brain*.

[31] Weintraub D., Doshi J., Koka D. (2011). Neurodegeneration across stages of cognitive decline in Parkinson disease. *Archives of Neurology*.

[32] Burton E. J., McKeith I. G., Burn D. J., Williams E. D., O'Brien J. T. (2004). Cerebral atrophy in Parkinson's disease with and without dementia: a comparison with Alzheimer's disease, dementia with Lewy bodies and controls. *Brain*.

[33] Summerfield C., Junqué C., Tolosa E. (2005). Structural brain changes in parkinson disease with dementia: a voxel-based morphometry study. *Archives of Neurology*.

[34] Nagano-Saito A., Washimi Y., Arahata Y. (2005). Cerebral atrophy and its relation to cognitive impairment in Parkinson disease. *Neurology*.

[35] Beyer M. K., Janvin C. C., Larsen J. P., Aarsland D. (2007). A magnetic resonance imaging study of patients with Parkinson's disease with mild cognitive impairment and dementia using voxel-based morphometry. *Journal of Neurology, Neurosurgery & Psychiatry*.

[36] Pereira J. B., Svenningsson P., Weintraub D. (2014). Initial cognitive decline is associated with cortical thinning in early Parkinson disease. *Neurology*.

[37] Agosta F., Canu E., Stefanova E. (2014). Mild cognitive impairment in Parkinson's disease is associated with a distributed pattern of brain white matter damage. *Human Brain Mapping*.

[38] Jubault T., Gagnon J.-F., Karama S. (2011). Patterns of cortical thickness and surface area in early Parkinson's disease. *NeuroImage*.

[39] Karagulle Kendi A. T., Lehericy S., Luciana M., Ugurbil K., Tuite P. (2008). Altered diffusion in the frontal lobe in Parkinson disease. *American Journal of Neuroradiology*.

[40] Husárová I., Mikl M., Lungu O. V., Mareček R., Vaníček J., Bareš M. (2013). Similar circuits but different connectivity patterns between the cerebellum, basal ganglia, and supplementary motor area in early Parkinson's disease patients and controls during predictive motor timing. *Journal of Neuroimaging*.

[41] Theilmann R. J., Reed J. D., Song D. D. (2013). White-matter changes correlate with cognitive functioning in Parkinson's disease. *Frontiers in Neurology*.

[42] Huang C., Mattis P., Tang C., Perrine K., Carbon M., Eidelberg D. (2007). Metabolic brain networks associated with cognitive function in Parkinson's disease. *NeuroImage*.

[43] Hanganu A., Bedetti C., Jubault T. (2013). Mild cognitive impairment in patients with Parkinson's disease is associated with increased cortical degeneration. *Movement Disorders*.

[44] MacDonald V., Halliday G. M. (2002). Selective loss of pyramidal neurons in the pre-supplementary motor cortex in Parkinson's disease. *Movement Disorders*.

[45] Kim J. P., Seo S. W., Shin H. Y. (2015). Effects of education on aging-related cortical thinning among cognitively normal individuals. *Neurology*.

[46] Ekman U., Eriksson J., Forsgren L., Mo S. J., Riklund K., Nyberg L. (2012). Functional brain activity and presynaptic dopamine uptake in patients with Parkinson's disease and mild cognitive impairment: a cross-sectional study. *The Lancet Neurology*.

[47] Monchi O., Stoessl A. J. (2012). Imaging neural correlates of mild cognitive impairment in Parkinson's disease. *The Lancet Neurology*.

[48] Monchi O., Degroot C., Mejia-Constain B., Bruneau M.-A. (2012). Neuroimaging studies of different cognitive profiles in Parkinson's disease. *Parkinsonism & Related Disorders*.

[49] Alexander G. E., DeLong M. R., Strick P. L. (1986). Parallel organization of functionally segregated circuits linking basal ganglia and cortex. *Annual Review of Neuroscience*.

[50] Alexander G. E., Crutcher M. D. (1990). Functional architecture of basal ganglia circuits: neural substrates of parallel processing. *Trends in Neurosciences*.

[51] Mehler-Wex C., Riederer P., Gerlach M. (2006). Dopaminergic dysbalance in distinct basal ganglia neurocircuits: Implications for the pathophysiology of parkinson's disease, schizophrenia and attention deficit hyperactivity disorder. *Neurotoxicity Research*.

[52] Stuss D. T., Levine B., Alexander M. P. (2000). Wisconsin Card Sorting Test performance in patients with focal frontal and posterior brain damage: effects of lesion location and test structure on separable cognitive processes. *Neuropsychologia*.

[53] MacPherson S. E., Phillips L. H., Della Sala S., Cantagallo A. (2009). Iowa Gambling task impairment is not specific to ventromedial prefrontal lesions. *Clinical Neuropsychologist*.

[54] Rogalsky C., Vidal C., Li X., Damasio H. (2012). Risky decision-making in older adults without cognitive deficits: an fMRI study of VMPFC using the Iowa Gambling Task. *Social Neuroscience*.

[55] Bechara A., Damasio H., Tranel D., Damasio A. R. (1997). Deciding advantageously before knowing the advantageous strategy. *Science*.

[56] Drevets W. C., Raichle M. E., Gazzaniga M. S. (1995). Positron emission tomographic imaging studies of human emotional disorders. *The Cognitive Neurosciences*.

[57] Cools R. (2006). Dopaminergic modulation of cognitive function-implications for L-DOPA treatment in Parkinson's disease. *Neuroscience & Biobehavioral Reviews*.

[58] Shohamy D., Myers C. E., Grossman S., Sage J., Gluck M. A., Poldrack R. A. (2004). Cortico-striatal contributions to feedback-based learning: converging data from neuroimaging and neuropsychology. *Brain*.

[59] Brand M., Labudda K., Kalbe E. (2004). Decision-making impairments in patients with Parkinson's disease. *Behavioural Neurology*.

[60] Segura B., Baggio H. C., Marti M. J. (2014). Cortical thinning associated with mild cognitive impairment in Parkinson's disease. *Movement Disorders*.

[61] Ibarretxe-Bilbao N., Junque C., Segura B. (2012). Progression of cortical thinning in early Parkinson's disease. *Movement Disorders*.

[62] Du A.-T., Schuff N., Kramer J. H. (2007). Different regional patterns of cortical thinning in Alzheimer's disease and frontotemporal dementia. *Brain*.

[63] Korf E. S. C., Wahlund L.-O., Visser P. J., Scheltens P. (2004). Medial temporal lobe atrophy on MRI predicts dementia in patients with mild cognitive impairment. *Neurology*.

[64] Abe Y., Kachi T., Kato T. (2003). Occipital hypoperfusion in Parkinson's disease without dementia: correlation to impaired cortical visual processing. *Journal of Neurology Neurosurgery and Psychiatry*.

[65] Nagamachi S., Wakamatsu H., Kiyohara S. (2008). Usefulness of rCBF analysis in diagnosing Parkinson's disease: supplemental role with MIBG myocardial scintigraphy. *Annals of Nuclear Medicine*.

[66] Bohnen N. I., Minoshima S., Giordani B., Frey K. A., Kuhl D. E. (1999). Motor correlates of occipital glucose hypometabolism in Parkinson’s disease without dementia. *Neurology*.

[67] Fénelon G., Mahieux F., Huon R., Ziégler M. (2000). Hallucinations in Parkinson's disease: prevalence, phenomenology and risk factors. *Brain*.

[68] Holroyd S., Currie L., Wooten G. F. (2001). Prospective study of hallucinations and delusions in Parkinson's disease. *Journal of Neurology Neurosurgery & Psychiatry*.

[69] Pappert E. J., Goetz C. G., Niederman F. G., Raman R., Leurgans S. (1999). Hallucinations, sleep fragmentation, and altered dream phenomena in Parkinson's disease. *Movement Disorders*.

[70] Sanchez-Ramos J. R., Ortoll R., Paulson G. W. (1996). Visual hallucinations associated with Parkinson disease. *Archives of Neurology*.

[71] Ramírez-Ruiz B., Martí M.-J., Tolosa E. (2007). Cerebral atrophy in Parkinson's disease patients with visual hallucinations. *European Journal of Neurology*.

[72] Park H. K., Kim J. S., Im K. C. (2013). Visual hallucinations and cognitive impairment in Parkinson's disease. *Canadian Journal of Neurological Sciences*.

[73] Geng D.-Y., Li Y.-X., Zee C.-S. (2006). Magnetic resonance imaging-based volumetric analysis of basal ganglia nuclei and substantia nigra in patients with Parkinson's disease. *Neurosurgery*.

[74] Laakso M. P., Partanen K., Riekkinen P. (1996). Hippocampal volumes in Alzheimer's disease, Parkinson's disease with and without dementia, and in vascular dementia: an MRI study. *Neurology*.

[75] Lee H. M., Kwon K.-Y., Kim M.-J. (2014). Subcortical grey matter changes in untreated, early stage Parkinson's disease without dementia. *Parkinsonism & Related Disorders*.

[76] Monchi O., Hanganu A. (2015). Reply: is nucleus accumbens atrophy correlated with cognitive symptoms of Parkinson's disease?. *Brain*.

[77] Farley I. J., Price K. S., Hornykiewicz O. (1977). Dopamine in thelimbic regions of the human brain: normal and abnormal. *Advances in Biochemical Psychopharmacology*.

[78] Kish S. J., Shannak K., Hornykiewicz O. (1988). Uneven pattern of dopamine loss in the striatum of patients with idiopathic Parkinson's disease. *The New England Journal of Medicine*.

[79] Stoeckel C., Gough P. M., Watkins K. E., Devlin J. T. (2009). Supramarginal gyrus involvement in visual word recognition. *Cortex*.

[80] Rektorova I., Mikl M., Barrett J., Marecek R., Rektor I., Paus T. (2012). Functional neuroanatomy of vocalization in patients with Parkinson's disease. *Journal of the Neurological Sciences*.

[81] Price C. J., Moore C. J., Humphreys G. W., Wise R. J. S. (1997). Segregating semantic from phonological processes during reading. *Journal of Cognitive Neuroscience*.

[82] Desikan R. S., Ségonne F., Fischl B. (2006). An automated labeling system for subdividing the human cerebral cortex on MRI scans into gyral based regions of interest. *NeuroImage*.

[83] Hoshi E. (2006). Functional specialization within the dorsolateral prefrontal cortex: a review of anatomical and physiological studies of non-human primates. *Neuroscience Research*.

[84] Mylius V., Ayache S. S., Ahdab R. (2013). Definition of DLPFC and M1 according to anatomical landmarks for navigated brain stimulation: inter-rater reliability, accuracy, and influence of gender and age. *NeuroImage*.

